# Relationships between Composition of Major Fatty Acids and Fat Distribution and Insulin Resistance in Japanese

**DOI:** 10.1155/2017/1567467

**Published:** 2017-04-30

**Authors:** Chikako Fujii, Toshihide Kawai, Koichiro Azuma, Yuko Oguma, Fuminori Katsukawa, Hiroshi Hirose, Kumiko Tanaka, Shu Meguro, Hideo Matsumoto, Hiroshi Itoh

**Affiliations:** ^1^Department of Internal Medicine, Keio University School of Medicine, Tokyo, Japan; ^2^Institute for Integrated Sports Medicine, Keio University School of Medicine, Tokyo, Japan; ^3^Department of Internal Medicine, Tokyo Saiseikai Central Hospital, Tokyo, Japan

## Abstract

*Objective*. The aim of this study was to evaluate the relationships between the composition of free fatty acids (FFAs) and metabolic parameters, including body fat distribution, in Japanese. *Methods*. The study subjects were 111 Japanese patients (54 males, 57 females). Metabolic parameters and visceral and subcutaneous fat areas as determined by CT scanning at the umbilical level were measured. Glucose tolerance test (GTT) was performed by administering 75 g glucose orally. *Results*. The percentage of linoleic acid (C18:2), the greatest constituent among FFAs, was negatively correlated with visceral fat area (*r* = −0.411, *p* < 0.0001), fasting glucose (*r* = −0.330, *p* < 0.0001), HbA1c (*r* = −0.231, *p* = 0.0146), and systolic blood pressure (*r* = −0.224, *p* = 0.0184). Linoleic acid percentage was also significantly negatively correlated with HOMA-IR (*r* = −0.416, *p* < 0.0001) by simple correlation. Based on the findings of OGTT, the 111 subjects were classified into three groups: 33 with normal glucose tolerance, 71 with impaired glucose tolerance (IGT), and 7 diabetic subjects. The percentage of serum linoleic acid in diabetic subjects was significantly lower than that in normal subjects. *Conclusion*. We conclude that serum linoleic acid level is negatively correlated with the accumulation of visceral fat in relation to a reduction of insulin resistance in Japanese subjects.

## 1. Introduction

Approximately 415 million people worldwide have diabetes mellitus [1], and the number is expected to increase to 642 million by the year 2040 [1, 2]. Diabetes mellitus is associated with a markedly increased risk of coronary heart disease, stroke, and renal failure, as well as disability [3]. Free fatty acids (FFAs), which are absorbed from ingested food and also liberated by adipocytes and reassembled into triglycerides, are often elevated in obese individuals. The accumulation of visceral fat induces insulin resistance and worsens glucose and lipid metabolism and is important in the development of type 2 diabetes [4]. Recently, the effects of FFAs on insulin's action have been investigated intensively [5, 6]. We have previously shown that FFAs cause *β* cell damage mainly by apoptosis in vitro [7] and by ER stress in mice [8]. The serum composition of FFAs in humans differs from race to race, and from environment to environment, because it reflects the average fat intake in the preceding days [9–12]. Despite intensive in vitro and in vivo studies to elucidate the impairment of insulin's action induced by chronic elevation of FFAs, however, the physiological interactions whereby the chronic effects of fatty acids on pancreatic beta cells affect insulin's action on glucose and insulin resistance remain unclear. To clarify these issues, firstly we evaluated the relationships between serum FFA composition and body fat area as determined by computed tomographic (CT) scanning at the umbilical level. Second, we evaluated the relationships between three major FFAs and the presence of diabetes, in humans.

## 2. Materials and Methods

This cross-sectional study was conducted in accordance with the Declaration of Helsinki and was approved by the Ethics Review Board of Keio University.

### 2.1. Participants

We enrolled 111 Japanese patients (54 men and 57 women) who attended our outpatient sports clinic at Keio University Hospital in Tokyo (Table 1). In men, mean ± standard error of the mean (SEM) age was 50.1 ± 1.4 years; body mass index (BMI), 26.7 ± 0.7 kg/m^2^; fasting plasma glucose (FPG), 109.2 ± 2.4 mg/dL; and hemoglobin A1c (HbA1c), 5.6 ± 0.2%. In women, mean ± SEM age was 52.7 ± 1.8 years; BMI, 26.2 ± 0.8 kg/m^2^; FPG, 104.9 ± 2.0 mg/dL; and HbA1c, 5.9 ± 0.1%. All subjects received dietary instructions for using a meal-exchange plan by nutritionists. The ideal dietary caloric intake for each patient was calculated as the ideal body weight (kilograms) × 25–30 kcal/kg. We recommended them to take 50–60% total calories as carbohydrate, 20–25% as fat, and 15–20% as protein. The subjects' meal preference did not deviate from that of the standard Japanese population. The physical activity level for each subject was determined by a questionnaire. None of the subjects was taking any medication or was a smoker. The nature of the procedure was explained to the subjects, and written informed consent was obtained from all participants.

### 2.2. Measurements and OGTT

The body weight of each patient was measured at the sports clinic. BMI was determined as weight corrected for height: weight (kg)/height (m^2^). Blood pressure (BP) was determined in the sitting position after a 10-minute rest.

A fasting blood sample was drawn from each subject before breakfast in the early morning, after an overnight fast. Glucose tolerance test (GTT) was performed by administering 75 g glucose orally, and the results were classified as normal glucose tolerance, impaired glucose tolerance (IGT), or diabetes on the basis of the World Health Organization criteria [13]. Plasma glucose (PG) was determined by the glucose oxidase method. Plasma immunoreactive insulin (IRI) level was measured by radioimmunoassay (Shionogi, Tokyo, Japan). Twenty-four fractions of FFAs (lauric, myristic, myristoleic, palmitic, palmitoleic, stearic, oleic, linoleic, gamma-linolenic, linolenic, arachidic, eicosenoic, eicosadienoic, 5-8-11-eicosatrienoic, dihomo-gamma-linolenic, arachidonic, eicosapentaenoic, behenic, erucic, docosatetraenoic, docosapentaenoic, lignoceric, docosahexaenoic, and nervonic acid) (Table 2) were determined by capillary gas chromatography (HP 6890, Hitachi, 30 m capillary column, inner diameter 0.32 mm, phase layer 0.25 *μ*m) [14]. The n6/n3 polyunsaturated fatty acid (PUFA) ratio was calculated as (linoleic + gamma linolenic + eicosadienoic + dihomo-gamma-linolenic + arachidonic + docosatetraenoic acid) / (linolenic + eicosapentaenoic + docosapentaenoic + docosahexaenoic acids). Glycosylated hemoglobin (HbA1c) was determined by high-performance liquid chromatography (HPLC) (Toso Co. Ltd., Tokyo, Japan). Total cholesterol (TC), high-density lipoprotein cholesterol (HDL-C), low-density lipoprotein cholesterol (LDL-C), and triglyceride (TG) were measured enzymatically (Hitachi autoanalyzer). Insulin resistance was assessed using the homeostasis model assessment (HOMA) system described by Matthews et al. [15] with the formula (insulin  (*μ*U/mL) × plasma  glucose  (mg/dL)/405). High molecular weight (HMW) adiponectin was determined by sensitive latex particle-enhanced turbidimetric immunoassay (LTIA) (Hitachi H7170, Japan). Leptin was determined by radioimmunoassay (Hitachi ARC-950, Japan).

### 2.3. Computed Tomography

Subcutaneous and visceral fat distributions were determined by measuring a −150 to −50 Hounsfield unit (HU) area using CT scanning at the umbilical level as described previously [16]. V/S ratio was calculated as visceral fat area/subcutaneous fat area.

### 2.4. Statistical Analysis

All results are expressed as mean ± SEM. Relationships between variables were analyzed by simple correlation and by linear stepwise regression analysis with calculation of Pearson product correlation coefficients. The Kruskal-Wallis test was used to evaluate comparisons among groups. A *p* value less than 0.05 was considered statistically significant. Statistical analyses were carried out using SPSS Statistics 23 software (IBM, Armonk, NY, USA).

## 3. Results

### 3.1. Relationships between FFA Composition and Metabolic Parameters

We measured twenty-four fractions of FFAs in all subjects, as shown in Table 2. Mean ± SEM proportions of constituents were 29.7 ± 0.5% linoleic acid, 22.4 ± 0.2% palmitic acid, 20.1 ± 0.3% oleic acid, 6.9 ± 0.1% stearic acid, 5.6 ± 0.1% arachidonic acid, and 4.6 ± 0.2% docosahexaenoic acid, and the others were below 3.0% each. First, we analyzed the relationships between twenty-four FFA fractions and twenty-two metabolic parameters (BMI, sBP, dBP, 0 min glucose, 120 min glucose, 0 min IRI, 120 min IRI, HbA1c, HOMA-IR, I.I, TC, TG, HDL-C, LDL-C, aspartate aminotransferase (AST), alanine aminotransferase (ALT), creatinine, uric acid, HMW adiponectin, leptin, visceral fat area, and subcutaneous fat area) by simple correlation analysis (Supplementary Table available online at https://doi.org/10.1155/2017/1567467). Second, multivariate analysis models were developed by stepwise regression with twenty-two metabolic parameters as dependent variables and by simple correlation with significant fractions among all twenty-four FFA fractions as independent variables.  Table 3 shows the relationships between dependent metabolic variables and the most significant independent FFA variables.

**Figure 1:**
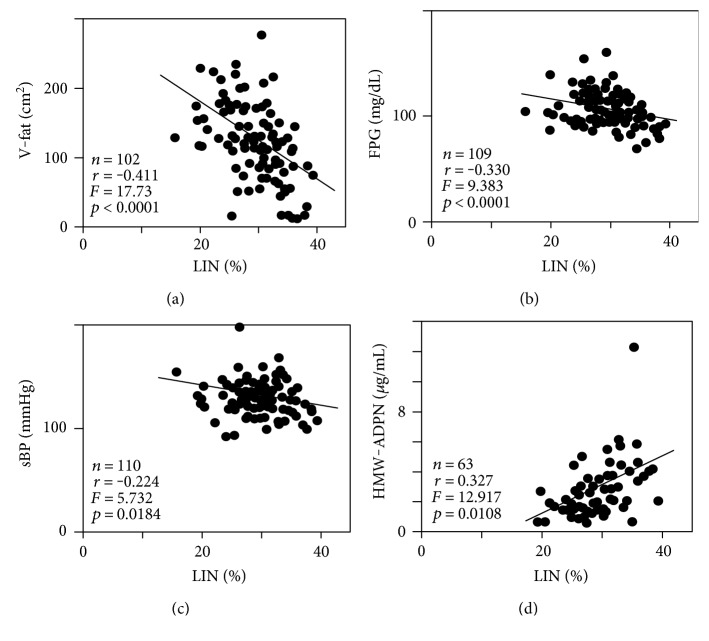
Relationships between ratio of serum linoleic acid (LIN, C18:2) and that of visceral fat area (V-fat) as determined by computed tomographic (CT) scanning at the umbilical level (a), that of fasting plasma glucose (FPG) (b), that of systolic blood pressure (sBP) (c), and that of high molecular weight (HMW) adiponectin (ADPN) (d) by linear stepwise regression analysis.

**Figure 2:**
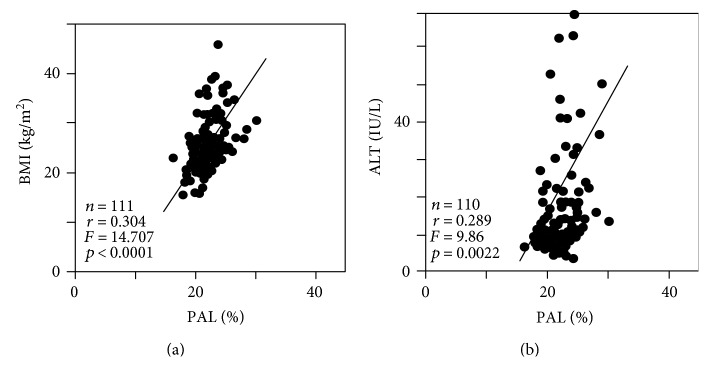
Relationships between ratio of serum palmitic acid (PAL, C16:0) and that of body mass index (BMI) (a) and that of alanine aminotransferase (ALT) (b).

**Figure 3:**
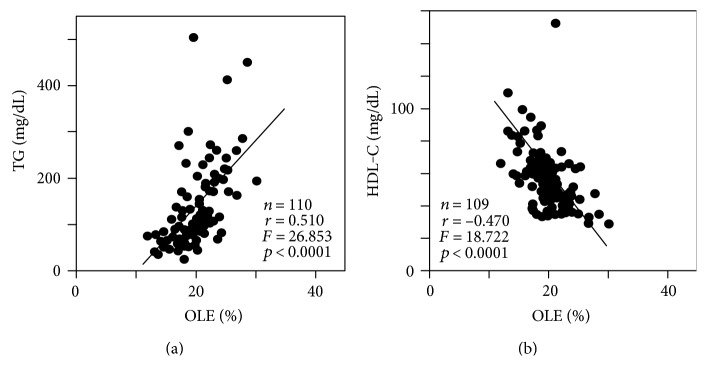
Relationships between ratio of serum oleic acid (OLE, C18:1) and that of triglyceride (TG) (a) and that of high-density lipoprotein cholesterol (HDL-C) (b).

As the most significant contributory cause, linoleic acid (%), the greatest constituent among the twenty-four FFA fractions, was negatively correlated with visceral fat area (*r* = −0.411, *F* = 17.73, *p* < 0.0001) (Figure 1(a)), whereas it was not correlated with subcutaneous fat area. Linoleic acid was negatively correlated with FPG (*r* = −0.330, *F* = 9.383, *p* < 0.0001) (Figure 1(b)) and sBP (*r* = −0.224, *F* = 5.732, *p* = 0.0184) (Figure 1(c)) and positively correlated with HMW adiponectin (*n* = 63, *r* = 0.327, *F* = 12.917, *p* = 0.0108) (Figure 1(d)). In addition, linoleic acid was also significantly negatively correlated with 120 min IRI (*r* = −0.383, *p* < 0.0001) and HOMA-IR (*r* = −0.416, *p* < 0.0001) by simple correlation analysis (Supplementary Table). Palmitic acid (%) was positively correlated with BMI (*r* = 0.304, *F* = 14.707, *p* < 0.0001) (Figure 2(a)) and ALT (*r* = 0.289, *F* = 9.86, *p* = 0.0022) (Figure 2(b)). Oleic acid (%) was positively correlated with TG (*r* = 0.510, *F* = 26.853, *p* < 0.0001) (Figure 3(a)) and negatively correlated with HDL-C (*r* = −0.470, *F* = 18.722, *p* < 0.0001) (Figure 3(b)).

### 3.2. OGTT and Fraction of Three Major FFAs

**Figure 4:**
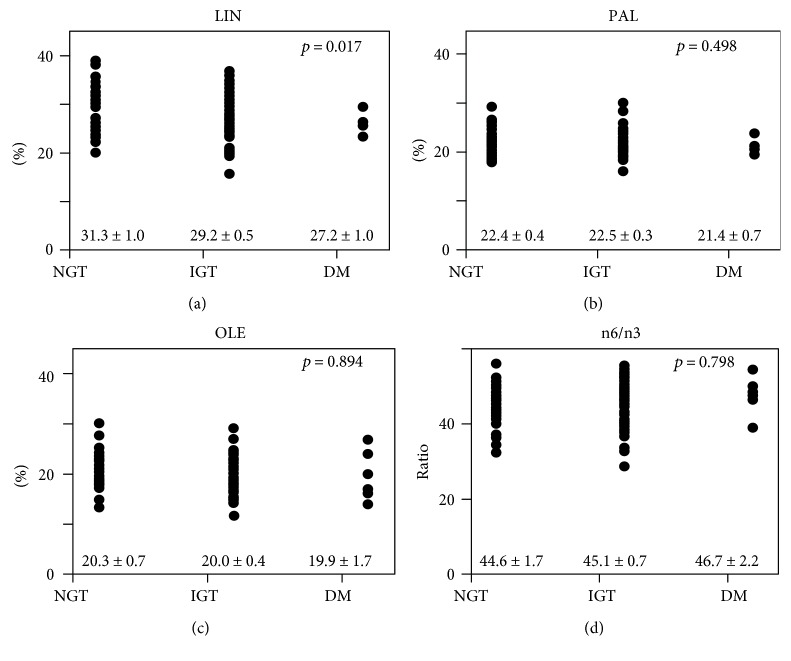
Percentages of linoleic acid (C18:2) (a), palmitic acid (C16:0) (b), and oleic acid (C18:1) (c) and n6/n3 PUFA ratio (d) in serum in normal glucose tolerant (NGT, *n* = 33), impaired glucose tolerant (IGT, *n* = 71), and diabetic (DM, *n* = 7) Japanese subjects by a cross-sectional evaluation. Kruskal-Wallis test was performed. Data are mean ± SEM.

Based on the findings of OGTT, the 111 subjects were classified into three groups: 33 with normal glucose tolerance, 71 with IGT, and 7 diabetic subjects (Table 4). There were no significant differences in macronutrient balance and physical activity level among the three groups.  Figure 4 shows the relationships between the major three FFAs and classification by OGTT. The percentage of linoleic acid in diabetic subjects was significantly lower than that in normal subjects (Figure 4(a)). On the other hand, the percentages of palmitic acid (Figure 4(b)) and oleic acid (Figure 4(c)) showed no significant differences among the groups. The n6/n3 PUFA ratio was not significantly different among the groups (Figure 4(d)).

**Figure 5:**
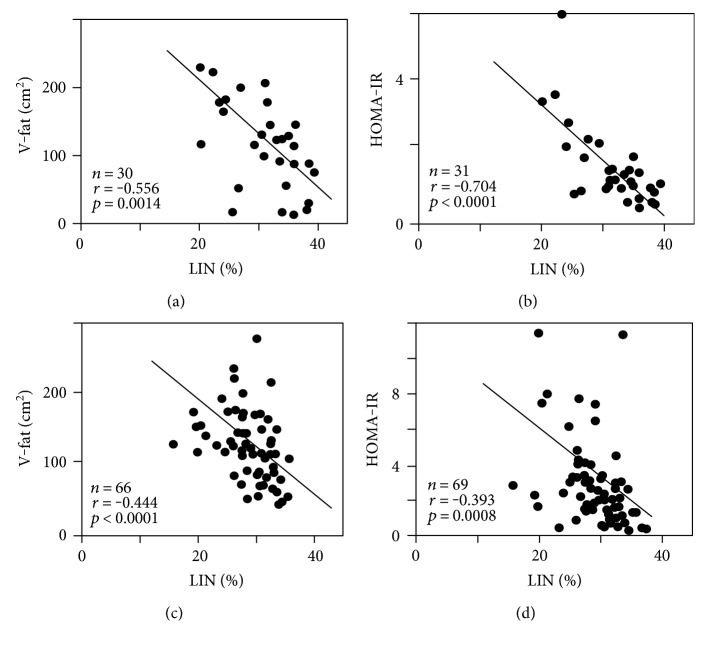
Relationships between ratio of serum linoleic acid (LIN, C18:2) and that of visceral fat area (V-fat) (a) and that of homeostasis model assessment insulin resistance index (HOMA-IR) (b) in subjects with normal glucose tolerance and ratio of serum linoleic acid and that of V-fat (c) and that of HOMA-IR (d) in subjects with impaired glucose tolerance, by simple correlation analysis.

Linoleic acid was significantly negatively correlated with visceral fat area (*n* = 30, *r* = −0.556, *p* = 0.0014) (Figure 5(a)) and HOMA-IR (*n* = 31, *r* = −0.704, *p* < 0.0001) (Figure 5(b)) by simple correlation analysis in normal subjects. In IGT subjects, linoleic acid was also significantly negatively correlated with visceral fat area (*n* = 66, *r* = −0.444, *p* < 0.0001) (Figure 5(c)) and HOMA-IR (*n* = 69, *r* = −0.393, *p* = 0.0008) (Figure 5(d)), as well as in normal subjects. The percentage of linoleic acid in IGT subjects with insulin resistance, which was defined as HOMA-IR > 2.5, was significantly lower than that in normal subjects (linoleic acid (%); 31.32 ± 0.97 in NGT (*n* = 30) versus 27.58 ± 0.76 in IGT with HOMA-IR > 2.5 (*n* = 30), *p* = 0.0036). In addition, the percentages of linoleic acid in IGT subjects with HOMA-IR > 2.5 were significantly lower than those in IGT subjects with HOMA-IR < 2.5 (linoleic acid (%); 30.45 ± 0.93 in IGT with HOMA-IR < 2.5 (*n* = 41) versus 27.58 ± 0.76 in IGT with HOMA-IR > 2.5 (*n* = 30), *p* = 0.0051).

## 4. Discussion

To investigate which FFA fraction is beneficial or deleterious for the development of type 2 diabetes in a race is important. In this study, we demonstrated that serum linoleic acid (%) was negatively correlated with the accumulation of visceral fat in Japanese subjects by a cross-sectional evaluation. Linoleic acid, which is an essential fatty acid, is the greatest fatty acid component in serum in Japanese subjects. The percentage of linoleic acid was negatively correlated with visceral fat area and was significantly lower in diabetic subjects than in normal subjects in our study.

This observation in our study is consistent with a report that compared the risk of developing type 2 diabetes in relation to FFA fractions in serum in a longitudinal observation of Finnish subjects [17]. In addition, the percentage of linoleic acid was negatively correlated with visceral fat area in our study. Because the accumulation of visceral fat worsens glucose and lipid metabolism and is important in the development of type 2 diabetes [4], a low level of linoleic acid in serum might be related to the development of type 2 diabetes. However, our cross-sectional evaluation in humans raises concern that the causal relation between linoleic acid and visceral fat remains unknown.

It is reported that dietary safflower oil, which is rich in the linoleic acid, reduced trunk adipose mass and increased total body lean mass in obese women with type 2 diabetes and was associated with gene expression of uncoupling protein (UCP) 1 and UCP content in the adipose tissue of rats [18, 19]. Park et al. [20] showed that a diet supplemented with conjugated linoleic acid decreased body weight in mice and enhanced fatty acid *β*-oxidation, supported by increased carnitine palmitoyltransferase activity in the skeletal muscle and fat pad, and also that linoleic acid reduced lipoprotein lipase activity while apparently enhancing lipolysis in a study of 3T3-L1 adipocytes treated with conjugated linoleic acid. In the present study, including subjects without diabetes, the percentage of linoleic acid was significantly negatively correlated with visceral fat area. Although we cannot exclude that the reduced body weight by linoleic acid in an animal study was due to a toxic effect on adipocytes, it is rational that visceral fat does not cause a decrease in linoleic acid level in serum, but linoleic acid decreases visceral fat in humans.

Adiponectin is generally present in plasma at a high concentration and is inversely associated with visceral fat accumulation [21]. High molecular weight form of adiponectin is considered an active form of adiponectin in vitro [22, 23]. Clinical studies [24, 25] also suggest that HMW adiponectin is more useful as an indicator than is total adiponectin, particularly in type 2 diabetic patients. We also conducted a cross-sectional study in healthy Japanese male subjects without any medication and reported that HMW adiponectin measured by ELISA was as effective as the HMW/total adiponectin ratio for predicting insulin resistance and/or metabolic syndrome [26]. Serum linoleic acid was correlated with HMW adiponectin in our study. It is suggested that linoleic acid and linoleic acid-derived fatty acids might increase serum adiponectin level through Keto A, which is a linoleic acid-derived fatty acid produced by gut lactic acid bacteria, and induce adipocyte differentiation via the activation of peroxisome proliferator-activated receptor γ (PPAR-γ) [27, 28]. This mechanism might be associated with the finding that pioglitazone, a PPAR-γ agonist, induced an increase in serum adiponectin level in humans [29].

Palmitic acid, which is the second major component in Japanese subjects, was correlated with BMI and ALT (Figure 2). Previously, we have shown that a palmitic acid-supplemented diet might cause deterioration of glucose tolerance by suppression of insulin secretion from pancreatic *β* cells in mice [8]. However, there was no difference in the level of palmitic acid among the normal, IGT, and diabetic groups in the current human study (Figure 4). It is reported that a palmitic acid-rich diet might change the gut microbiota and induce weight gain and hepatic lipid accumulation compared to an unsaturated fat diet in mice [30–32]. The discrepancies in the reported findings might be due to species differences.

Oleic acid, which is the third major FFA component in Japanese subjects, was correlated with serum TG and negatively correlated with HDL-C (Figure 3), while it was not correlated with parameters associated with insulin. In addition, there was no difference in oleic acid level among the normal, IGT, and diabetic groups (Figure 4). It is widely regarded that a Mediterranean diet, which is rich in oleic acid (olive oil), might be beneficial for the prevention and treatment of type 2 diabetes [33, 34]. On the other hand, in a recent study, oleic acid plasma level was shown to be a selective biomarker of impaired glucose tolerance in several cohorts [35]. These discrepancies might be related to racial differences. Investigation of the appropriate oleic acid intake in different races might be important.

The n6/n3 PUFA ratio is suggested to be important in disease development, for example, cardiovascular disease in rodent models fed with a high-fat diet [36]. However, the relationships of n6/n3 PUFA ratio and type 2 diabetes are not clear. In the present study, n6/n3 PUFA ratio was not significantly different among the normal, IGT, and diabetes groups (Figure 4(d)).

This study has several limitations. First, the sample size was small. Second, the study design did not allow for correlations between the dietary components of FFAs and the serum components of FFAs. Third, this cross-sectional study design raises concern that the causal relation between fatty acids and parameters remains unknown, as we discussed above. Further longitudinal studies are needed with a greater number of subjects. Lastly, the precise mechanisms for the results in this cross-sectional study should be elucidated. Recently, several studies have suggested that differences in the dietary composition might change the gut microbiota in obesity and type 2 diabetes [37–39]. de Wit et al. [32] showed that a diet rich in unsaturated fat induced changes in gut microbiota composition and mucosal PPARα target gene expression. Dietary *trans*-10, *cis*-12-conjugated linoleic acid (*t*10*c*12-CLA) significantly decreased visceral fat mass in mice. Analysis of the microbiota composition under *t*10*c*12-CLA supplementation revealed a lower proportion of Firmicutes and a higher proportion of Bacteroidetes compared with that under no supplementation [40]. Linoleic acid might change the gut microbiota composition and reduce visceral fat.

## 5. Conclusion

In conclusion, we demonstrated that linoleic acid percentage was negatively correlated with the accumulation of visceral fat in relation to a reduction of insulin resistance in Japanese subjects.

## Supplementary Material

Supplementary Table. Simple regression analysis of relationships between twenty-four FFA fractions and major variables (n=111). Correlation coefficients are shown.

## Figures and Tables

**Table 1:** Baseline characteristics of subjects.

Parameters	Values
*N* (male/female)	111 (54/57)
Age (years)	51.5 ± 1.1
Height (cm)	162.5 ± 0.8
Body mass index (kg/m^2^)	26.4 ± 0.5
Waist circumference (cm)	91.3 ± 1.4
Systolic blood pressure (mmHg)	128.4 ± 2.3
Diastolic blood pressure (mmHg)	78.2 ± 1.5
Biochemical markers	
Fasting plasma glucose (mg/dL)	107.0 ± 1.5
Hemoglobin A1c (%)	5.7 ± 0.9
Immunoreactive insulin (*μ*U/mL)	9.4 ± 0.7
Total cholesterol (mg/dL)	211.0 ± 5.9
High-density lipoprotein cholesterol (mg/dL)	57.1 ± 1.8
Low-density lipoprotein cholesterol (mg/dL)	123.8 ± 4.4
Triglyceride (mg/dL)	131.4 ± 8.0
Free fatty acids (mEq/L)	0.6 ± 0.0
Aspartate aminotransferase (IU/L)	26.2 ± 1.1
Alanine aminotransferase (IU/L)	31.9 ± 2.5
Creatinine (mg/dL)	0.7 ± 0.0
Uric acid (mg/dL)	5.8 ± 0.1
High molecular weight adiponectin (*μ*g/mL) (*n* = 63)	2.9 ± 0.2
Leptin (ng/mL) (*n* = 63)	12.4 ± 1.5
75 g OGTT	
30 min glucose (mg/dL)	180.1 ± 3.4
60 min glucose (mg/dL)	199.9 ± 5.3
120 min glucose (mg/dL)	161.9 ± 5.1
30 min IRI (*μ*U/mL)	50.4 ± 3.7
60 min IRI (*μ*U/mL)	70.1 ± 5.3
120 min IRI (*μ*U/mL)	68.7 ± 4.7
HOMA-IR	2.4 ± 0.2
Insulinogenic index	0.7 ± 0.1
CT scan	
Visceral fat (cm^2^)	122.6 ± 5.4
Subcutaneous fat (cm^2^)	209.0 ± 12.9
V/S ratio	0.7 ± 0.0

Data are mean ± SEM. IRI: immunoreactive insulin; HOMA-IR: homeostasis model assessment insulin resistance index.

**Table 2:** Component ratio analysis of twenty-four FFA fractions.

Free fatty acid	Symbol	%
Lauric acid	C12:0	0.098 ± 0.91
Myristic acid	C14:0	1.04 ± 0.40
Myristoleic acid	C14:1	0.098 ± 0.04
Palmitic acid	C16:0	22.37 ± 0.23
Palmitoleic acid	C16:1	2.57 ± 0.10
Stearic acid	C18:0	6.86 ± 0.10
Oleic acid	C18:1	20.06 ± 0.32
Linoleic acid	C18:2	29.73 ± 0.45
Gamma-linolenic acid	C18:3(6)	0.31 ± 0.16
Linolenic acid	C18:3(3)	0.88 ± 0.26
Arachidic acid	C20:0	0.051 ± 0.002
Eicosenoic acid	C20:1	0.19 ± 0.01
Eicosadienoic acid	C20:2	0.19 ± 0.01
5-8-11-Eicosatrienoic acid	C20:3(9)	0.054 ± 0.003
Dihomo-gamma-linolenic acid	C20:3(6)	1.19 ± 0.03
Arachidonic acid	C20:4	5.64 ± 0.13
Eicosapentaenoic acid	C20:5	2.87 ± 0.19
Behenic acid	C22:0	0.11 ± 0.01
Erucic acid	C22:1	0.026 ± 0.003
Docosatetraenoic acid	C22:4	0.13 ± 0.01
Docosapentaenoic acid	C22:5	0.82 ± 0.03
Lignoceric acid	C24:0	0.044 ± 0.003
Docosahexaenoic acid	C22:6	4.61 ± 0.17
Nervonic acid	C24:1	0.11 ± 0.05

Data are mean ± SEM.

**Table 3:** Stepwise regression analysis of relationships between dependent variables and the most significant independent FFA variables.

Dependent variable	Contributory cause	Correlation coefficient	*F* value	*p* value
BMI (kg/m^2^)	Palmitic acid	*r* = 0.304	14.707	<0.0001
sBP (mmHg)	Linoleic acid	*r* = −0.224	5.732	0.0184
dBP (mmHg)	—	—	—	—
Biomarkers				
0 min glu (mg/dL)	Linoleic acid	*r* = −0.330	9.383	<0.0001
120 min glu (mg/dL)	Linoleic acid	*r* = −0.389	14.041	<0.0001
0 min IRI (*μ*U/mL)	Myristic acid	*r* = 0.430	14.014	<0.0001
120 min IRI (*μ*U/mL)	Palmitoleic acid	*r* = 0.342	12.237	0.0006
HbA1c (*N*) (%)	Linoleic acid	*r* = −0.231	6.165	0.0146
HOMA-IR	Myristic acid	*r* = 0.520	13.792	<0.0001
I.I.	—	—	—	—
TC (mg/dL)	Linolenic acid	*r* = 0.282	5.554	0.0216
TG (mg/dL)	Oleic acid	*r* = 0.510	26.853	<0.0001
HDL-C (mg/dL)	Oleic acid	*r* = −0.470	18.722	<0.0001
LDL-C (mg/dL)	Behenic acid	*r* = −0.344	6.584	0.0134
AST (IU/L)	Myristic acid	*r* = 0.270	8.467	0.0044
ALT (IU/L)	Palmitic acid	*r* = 0.289	9.86	0.0022
Cre (mg/dL)	Gamma linolenic acid	*r* = −0.215	6.022	0.0337
UA (mg/dL)	Eicosenoic acid	*r* = 0.247	6.002	0.0162
HMW-ADPN (*μ*g/mL) (*n* = 63)	Linoleic acid	*r* = 0.327	12.917	0.0108
Leptin (ng/mL) (*n* = 63)	Lauric acid	*r* = 0.303	6.157	0.0159
CT scan				
V-fat (cm^2^)	Linoleic acid	*r* = −0.411	17.73	<0.0001
S-fat (cm^2^)	Dihomo-*γ*-linolenic acid	*r* = 0.272	9.221	0.0002

BMI: body mass index; sBP: systolic blood pressure; dBP: diastolic blood pressure; 0 min glu: 0 min glucose; 120 min glu: 120 min glucose; 0 min IRI: 0 min immunoreactive insulin; 120 min IRI: 120 min-immunoreactive insulin; HbA1c: hemoglobin A1c; HOMA-IR: homeostasis model assessment insulin resistance index; I.I.: insulinogenic index; TC: total cholesterol; TG: triglyceride; HDL-C: high-density lipoprotein cholesterol; LDL-C: low-density lipoprotein cholesterol; AST: aspartate aminotransferase; ALT: alanine aminotransferase; Cre: creatinine; UA: uric acid; HMW-ADPN: high molecular weight adiponectin; V-fat: visceral fat area; S-fat: subcutaneous fat area.

**Table 4:** Subjects classified as normal glucose tolerant, impaired glucose tolerant (IGT), and diabetic based on OGTT.

Parameters	Normal	IGT	Diabetes	*p* value
*N*	33	71	7	—
(Male/female)	(13/20)	(43/28)	(2/5)	—
Age (years)	47.9 ± 2.2	52.5 ± 1.4	58.4 ± 3.6	0.0601
Body mass index (kg/m^2^)	26.2 ± 1.1	26.5 ± 0.7	26.4 ± 0.9	0.808
Fasting glucose (mg/dL)	93.8 ± 1.6	109.4 ± 1.5	141.6 ± 4.8	<0.0001
Hemoglobin A1c (%)	5.2 ± 0.2	6.0 ± 0.1	6.4 ± 0.1	<0.0001
HOMA-IR	1.5 ± 0.2	2.7 ± 0.3	4.1 ± 1.5	0.0011
Visceral fat area (cm^2^)	115.6 ± 11.5	124.0 ± 6.3	141.3 ± 17.3	0.712

Kruskal-Wallis test was used for comparisons among groups. Data are mean ± SEM. HOMA-IR: homeostasis model assessment insulin resistance index.
