# Supplementary material for: Relationships between Composition of Major Fatty Acids and Fat Distribution and Insulin Resistance in Japanese
Source: J Diabetes Res. 2017 Apr 30;2017:1567467. doi: 10.1155/2017/1567467 (PMC5429966; doi:10.1155/2017/1567467)
Supplement: Supplementary file 1 — Supplementary Table. Simple regression analysis of relationships between twenty-four FFA fractions and major variables (n=111). Correlation coefficients are shown. [file 1567467.f1.docx]

**Supplementary table.** Simple regression analysis of relationships between twenty-four FFA fractions and major variables (n=111). Correlation coefficients are shown.

| **Free fatty acid** | BMI | sBP | dBP | 0min-glu | 120min-glu | 0min-IRI | **120min-IRI** |
| --- | --- | --- | --- | --- | --- | --- | --- |
| Lauric acid | 0.257* | -0.014 | -0.079 | -0.012 | 0.031 | 0.340* | 0.302* |
| Myristic acid | 0.225* | 0.055 | -0.010 | 0.218* | 0.166 | 0.476* | 0.378* |
| Myristoleic acid | -0.112 | -0.149 | -0.162 | -0.261* | -0.282* | -0.102 | -0.051 |
| Palmitic acid | 0.457* | 0.128 | 0.123 | -0.005 | 0.040 | 0.443* | 0.301* |
| Palmitoleic acid | 0.343* | 0.110 | 0.036 | 0.084 | 0.146 | 0.416* | 0.441* |
| Stearic acid | 0.063 | -0.056 | -0.048 | 0.148 | 0.056 | 0.251* | 0.200* |
| Oleic acid | 0.450* | 0.102 | 0.115 | -0.029 | 0.009 | 0.256* | 0.316* |
| Linoleic acid | -0.389* | -0.224* | -0.170 | -0.305* | -0.280* | -0.452* | -0.383* |
| Gamma linolenic acid | 0.329* | 0.061 | -0.030 | 0.178 | 0.131 | 0.320* | 0.197 |
| Linolenic acid | -0.183 | 0.016 | -0.038 | 0.034 | 0.106 | -0.013 | 0.182 |
| Arachidic acid | 0.081 | 0.063 | 0.144 | 0.063 | -0.014 | 0.058 | -0.041 |
| Eicosenoic acid | 0.043 | 0.155 | 0.184 | 0.226* | 0.203* | 0.017 | -0.048 |
| Eicosadienoic acid | -0.006 | 0.062 | 0.065 | 0.003 | 0.132 | -0.002 | 0.082 |
| 5-8-11 Eicosatrienoic acid | 0.287* | 0.034 | -0.051 | 0.129 | 0.079 | 0.310* | 0.235* |
| Dihomo-gamma linolenic acid | 0.358* | 0.029 | -0.054 | 0.125 | 0.113 | 0.341* | 0.390* |
| Arachidonic acid | -0.071 | -0.118 | -0.165 | 0.097 | -0.002 | -0.114 | -0.162 |
| Eicosapentaenoic acid | -0.287* | 0.157 | 0.142 | 0.242* | 0.190 | -0.173 | -0.225* |
| Behenic acid | -0.180 | -0.121 | -0.130 | 0.066 | -0.044 | 0.022 | -0.065 |
| Erucic acid | 0.246* | 0.058 | 0.096 | 0.159 | 0.237* | 0.172 | 0.146 |
| Docosatetraenoic acid | 0.294* | -0.063 | -0.120 | -0.054 | 0.013 | 0.239* | 0.255* |
| Docosapentaenoic acid | -0.408* | 0.055 | 0.039 | 0.136 | 0.142 | -0.210* | -0.175 |
| Lignoceric acid | -0.092 | 0.027 | 0.037 | 0.119 | 0.169 | -0.054 | -0.019 |
| Docosahexaenoic acid | -0.367* | 0.076 | 0.071 | 0.227* | 0.199* | -0.194* | -0.194* |
| Nervonic acid | -0.184 | 0.067 | 0.068 | 0.099 | 0.078 | -0.067 | -0.092 |

| **Free fatty acid** | HbA1c | HOMA-IR | **I.I.** | TC | TG | HDL-C | LDL-C |
| --- | --- | --- | --- | --- | --- | --- | --- |
| Lauric acid | 0.169 | 0.256* | -0.127 | -0.058 | 0.256* | -0.103 | -0.086 |
| Myristic acid | 0.161 | 0.436* | -0.093 | -0.050 | 0.499* | -0.243* | -0.119 |
| Myristoleic acid | 0.073 | -0.122 | -0.033 | -0.165 | -0.078 | 0.169 | 0.006 |
| Palmitic acid | 0.081 | 0.393* | -0.150 | -0.270* | 0.435* | -0.220* | -0.176 |
| Palmitoleic acid | 0.198* | 0.366* | -0.092 | -0.032 | 0.479* | -0.277* | 0.107 |
| Stearic acid | 0.153 | 0.198* | 0.185 | -0.068 | -0.019 | 0.051 | 0.121 |
| Oleic acid | 0.052 | 0.177 | -0.056 | -0.158 | 0.566* | -0.520* | -0.072 |
| Linoleic acid | -0.231* | -0.416* | 0.004 | 0.281* | -0.448* | 0.284* | 0.103 |
| Gamma linolenic acid | 0.124 | 0.265* | 0.029 | -0.224 | 0.118 | -0.209* | 0.014 |
| Linolenic acid | -0.088 | 0.016 | 0.044 | 0.297* | 0.221* | -0.097 | 0.087 |
| Arachidic acid | -0.060 | 0.075 | -0.036 | -0.107 | 0.127 | -0.036 | -0.152 |
| Eicosenoic acid | 0.041 | 0.077 | 0.137 | -0.125 | 0.106 | -0.146 | -0.164 |
| Eicosadienoic acid | -0.022 | 0.002 | 0.131 | 0.009 | 0.166 | -0.030 | 0.074 |
| 5-8-11 Eicosatrienoic acid | 0.094 | 0.292* | -0.108 | -0.105 | 0.188 | -0.238* | -0.006 |
| Dihomo-gamma linolenic acid | 0.091 | 0.273* | -0.046 | -0.234 | 0.135 | -0.279* | 0.111 |
| Arachidonic acid | 0.053 | -0.087 | 0.065 | -0.257* | -0.331* | 0.136 | -0.057 |
| Eicosapentaenoic acid | 0.046 | -0.092 | 0.092 | 0.062 | -0.323* | 0.292* | 0.062 |
| Behenic acid | -0.067 | 0.086 | 0.015 | -0.232 | -0.170 | 0.212* | -0.344* |
| Erucic acid | 0.231* | 0.132 | 0.071 | -0.027 | 0.242* | -0.215* | -0.056 |
| Docosatetraenoic acid | 0.004 | 0.223* | 0.085 | -0.254 | 0.192 | -0.218* | 0.078 |
| Docosapentaenoic acid | -0.151 | -0.147 | 0.110 | 0.107 | -0.119* | 0.335* | 0.024 |
| Lignoceric acid | 0.115 | -0.020 | 0.067 | -0.043 | <-0.001 | -0.017 | 0.108 |
| Docosahexaenoic acid | 0.064 | -0.116 | 0.105 | 0.062 | -0.319* | 0.348* | 0.022 |
| Nervonic acid | 0.079 | -0.019 | 0.045 | -0.029 | -0.180 | 0.099 | -0.013 |

| **Free fatty acid** | AST | **ALT** | **Cre** | **UA** | **HMW-ADPN** | Leptin | Vfat | Sfat |
| --- | --- | --- | --- | --- | --- | --- | --- | --- |
| Lauric acid | 0.154 | 0.125 | -0.221* | -0.109 | -0.262* | 0.303* | 0.143 | 0.233* |
| Myristic acid | 0.270* | 0.276* | -0.149 | -0.090 | -0.397* | 0.221 | 0.311* | 0.189 |
| Myristoleic acid | -0.112 | -0.118 | 0.068 | 0.054 | 0.081 | -0.035 | -0.272* | -0.096 |
| Palmitic acid | 0.187 | 0.289* | -0.008 | 0.113 | -0.470* | 0.242 | 0.372* | 0.237* |
| Palmitoleic acid | 0.250* | 0.275* | -0.057 | -0.041 | -0.310* | 0.284* | 0.411* | 0.295* |
| Stearic acid | 0.024 | -0.010 | -0.192* | -0.158 | -0.149 | 0.257* | 0.021 | 0.140 |
| Oleic acid | 0.109 | 0.255* | 0.113 | 0.082 | -0.352* | 0.236 | 0.427* | 0.248* |
| Linoleic acid | -0.211* | -0.256* | -0.024 | -0.146 | 0.480* | -0.242 | -0.504* | -0.270* |
| Gamma linolenic acid | 0.013 | 0.085 | -0.265* | -0.184 | -0.336* | 0.141 | 0.269* | 0.260* |
| Linolenic acid | 0.132 | 0.097 | <-0.001 | -0.159 | <-0.001 | -0.085 | 0.028 | -0.079 |
| Arachidic acid | -0.049 | -0.008 | 0.117 | 0.042 | 0.016 | -0.095 | 0.003 | 0.051 |
| Eicosenoic acid | 0.032 | 0.074 | 0.278* | 0.247* | 0.027 | -0.029 | 0.248* | 0.052 |
| Eicosadienoic acid | -0.021 | -0.074 | 0.052 | -0.031 | -0.154 | 0.108 | -0.037 | -0.001 |
| 5-8-11  Eicosatrienoic acid | 0.154 | 0.164 | -0.047 | -0.064 | -0.388* | 0.238 | 0.315* | 0.311* |
| Dihomo-gamma linolenic acid | 0.118 | 0.206* | -0.146 | -0.127 | -0.314* | 0.301* | 0.288* | 0.274* |
| Arachidonic acid | -0.154 | -0.190* | -0.071 | 0.014 | 0.107 | -0.011 | -0.159 | 0.014 |
| Eicosapentaenoic acid | -0.060 | -0.154 | 0.010 | 0.117 | 0.150 | -0.203 | -0.098 | -0.188 |
| Behenic acid | -0.033 | -0.064 | 0.043 | -0.028 | -0.079 | -0.120 | -0.112 | -0.089 |
| Erucic acid | 0.023 | 0.082 | -0.016 | 0.091 | -0.293* | 0.287* | 0.260* | 0.190 |
| Docosatetraenoic acid | 0.055 | 0.093 | 0.016 | 0.001 | -0.264* | 0.159 | 0.266* | 0.267* |
| Docosapentaenoic acid | 0.008 | -0.144 | 0.135 | 0.172 | 0.147 | -0.282* | -0.146 | -0.290* |
| Lignoceric acid | -0.086 | -0.074 | 0.128 | 0.141 | -0.068 | -0.140 | -0.052 | -0.065 |
| Docosahexaenoic acid | 0.006 | -0.154 | 0.100 | 0.114 | 0.242 | -0.263* | -0.160 | -0.215* |
| Nervonic acid | 0.006 | -0.037 | 0.124 | 0.218* | 0.044 | -0.209 | -0.155 | -0.166 |

p<0.05 * was considered statistically significant.

BMI, body mass index; sBP, systolic blood pressure; dBP, diastolic blood pressure; 0min-glu, 0min-glucose; 120min-glu, 120min-glucose; 0min-IRI, 0min-immunoreactive insulin; 120min-IRI, 120min-immunoreactive insulin; HbA1c, hemoglobin A1c; HOMA-IR, homeostasis model assessment insulin resistance index; I.I., insulinogenic index; TC, total cholesterol; TG, triglyceride; HDL-C, high-density lipoprotein cholesterol; LDL-C, low-density lipoprotein cholesterol; AST, aspartate aminotransferase; ALT, alanine aminotransferase; Cre, creatinine; UA, uric acid; HMW-ADPN, high molecular weight adiponectin; V fat, visceral fat area; S fat, subcutaneous fat area.
